# Interfacial effect on physical properties of composite media: Interfacial volume fraction with non-spherical hard-core-soft-shell-structured particles

**DOI:** 10.1038/srep16003

**Published:** 2015-11-02

**Authors:** Wenxiang Xu, Qinglin Duan, Huaifa Ma, Wen Chen, Huisu Chen

**Affiliations:** 1 Institute of Soft Matter Mechanics, College of Mechanics and Materials, Hohai University, Nanjing, China; 2 State Key Laboratory of Structural Analysis for Industrial Equipment, Dalian University of Technology, Dalian, China; 3 State Key Laboratory of Simulation and Regulation of Water Cycle in River Basin, China Institute of Water Resources and Hydropower Research, Beijing, China; 4 School of Materials Science and Engineering, Southeast University, Nanjing, China

## Abstract

Interfaces are known to be crucial in a variety of fields and the interfacial volume fraction dramatically affects physical properties of composite media. However, it is an open problem with great significance how to determine the interfacial property in composite media with inclusions of complex geometry. By the stereological theory and the nearest-surface distribution functions, we first propose a theoretical framework to symmetrically present the interfacial volume fraction. In order to verify the interesting generalization, we simulate three-phase composite media by employing hard-core-soft-shell structures composed of hard mono-/polydisperse non-spherical particles, soft interfaces, and matrix. We numerically derive the interfacial volume fraction by a Monte Carlo integration scheme. With the theoretical and numerical results, we find that the interfacial volume fraction is strongly dependent on the so-called geometric size factor and sphericity characterizing the geometric shape in spite of anisotropic particle types. As a significant interfacial property, the present theoretical contribution can be further drawn into predicting the effective transport properties of composite materials.

Interfaces are crucial components that can be found in a wide variety of fields like soft matter systems, self-assembled structures, and composite materials^1^,^2^,^3^. In all these cases, materials are normally viewed as a three-phase composite consisting of mono-/polydisperse hard particles of anisotropic geometry, interfacial layers with a predefined dimension coating around hard particles, and matrix, which is known as the hard-core-soft-shell (HCSS) structure^3^,^4^,^5^,^6^,^7^,^8^. Also, the volume fraction of each constituent phase in HCSS structure has a distinct influence on effective properties of materials by effective medium approximations^9^,^10^,^11^. However, the interfacial phase is photographed to be essentially a complex network structure that neighboring interfacial layers possess an overlapping potential^6^,^7^, so that it is a practical challenge to capture the volume fraction of interfaces through experiments. Specifically, the geometric configurations of inclusions giving rise to a variation of the interfacial property have been systematically sought empirically, since it is expected to allow researchers to better understand behaviors of materials and to optimize their design.

Theoretical and numerical studies for the interfacial volume fraction have been attempted in the last two decades. It can be traced back to the late 20th century, the pioneering work from Lu and Torquato^12^ that developed a theory of the nearest-surface distribution functions to compute the so-called void “exclusion” probability. Thereafter, considerable attention has been paid to the application of such the theory to the interfacial volume fraction around spherical or ellipsoidal particles^13^,^14^,^15^. Additionally, the interfacial volume fraction around ellipsoidal particles was also evaluated numerically by the Monte Carlo simulations^14^,^15^. But the derived results are widely divergent. All these inconsistent results underscore the difficulty in determining the interfacial volume fraction around ellipsoidal particles. As a sophisticated approximation for hard cores, regular convex polyhedra have been extensively proposed to represent hard inclusions in the research of composite media^16^,^17^,^18^. Also, the interfacial volume fraction around polydisperse Platonic particles was recently presented^19^. In the above previous studies, the interfacial volume fraction around anisotropic particles is typically investigated on a case-by-case basis using computer simulations or approximate schemes, even though these predominant lines of research are of significance for understanding the interfacial volume fraction, there is yet no a theoretical framework to systematically estimate the interfacial volume fraction so far. Furthermore, how anisotropic morphological details affect the interfacial volume fraction remains dubious. It is our objective in this work to address this gap.

## Results

### Theoretical framework for interfacial volume fraction

We find that the theory of the nearest-surface distribution functions^12^ can be well applied to derive the interfacial volume fraction around anisotropic particles, the detailed derivation of which is presented in Methods section. The formulas of the interfacial volume fraction are given by


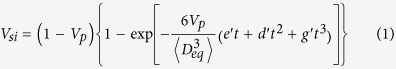


with


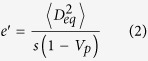



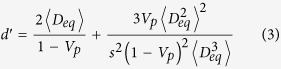



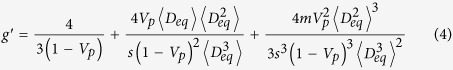


where *V*_*si*_ and *V*_*p*_ are the volume fractions of interfaces and particles, respectively. *D*_*eq*_ is an equivalent diameter defined as the diameter of a sphere having the same volume as an anisotropic particle^14^,^19^. 〈 〉 indicates a number-averaged treatment. *m* is a parameter, the value of which depends on the theoretical approximation of the radial distribution function of spherical particle systems, such as *m* = 0 being the Percus-Yevick approximation^20^, *m* = 2 being the Carnahan-Starling approximation^21^, and *m* = 3 being the scaled-particle approximation^22^. It is noted that the value of *m* does not seem to make much difference, since the term of equation (4) limited by the value of *m* is only a little contribution to the determination of the interfacial volume fraction (see Supplementary Information, Fig. S1). *t* and *s* are the interfacial dimension and sphericity defined as the ratio between the surface area of a sphere and that of a non-spherical particle with the same volume^23^, respectively, which is used as a morphological descriptor of anisotropic particles. In the whole particle system, sphericity of each particle is assigned to be identical so as to assess the impact of morphological details of anisotropic particles.

In order to account for the effects of geometric details of anisotropic particles, monodisperse particle structures should be considered. Accordingly, the parameters *e*^′^, *d*^′^, and *g*^′^ are expressed as


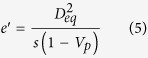



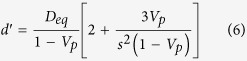



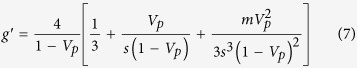


In the substitution of equations (5)–(7), 6, 7 into equation (1), and then let *λ* = *t*/*D*_*eq*_ (*λ* is defined as a geometric size factor of an anisotropic particle), *V*_*si*_ is rearranged by
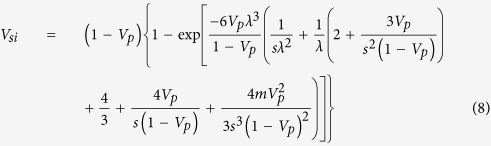


In accordance of the term of equation (8), the interfacial volume fraction of monodisperse anisotropic particle structures predominantly relies on the geometric size factor λ controlled by the coupling of interfacial dimension and equivalent diameter, except the sphericity of anisotropic particles. That suggests the constant λ with different *t* and *D*_*eq*_ possessing the uniform *V*_*si*_. It is an interesting generalization that can allow researchers to just control the geometric size factor λ to design the specific interfacial property in composite materials, instead of varying the interfacial dimension and the particle size described in the past. On the other hand, the investigation of polydisperse particle systems is illustrated in the Supplementary methods (S8). It is essentially stressed that the present contribution can also be extended to predict the mean spacing of complex geometric voids that is the topic in porous materials, even if the physical characteristic is not the focus of this work.

### Numerical experiments for interfacial volume fraction

We implement a series of numerical experiments to anisotropic particles of different types so as to verify the reliability of the theoretical scheme proposed above. Firstly, we simulate three-phase composite media by using the HCSS structure that is composed of hard anisotropic particles, soft interfaces, and matrix. Soft interfaces with a constant dimension are required to coat on the surface of each particle. The realistic morphology of such the interface can be realized by the Minkowski addition manner^24^,^25^, the details of which have been demonstrated in the recent study^25^. Fig. S2 displays some visualization examples for the construction of interfacial profiles around convex polyhedra by the Minkowski addition manner (see Supplementary Information, Fig. S2).

**Figure 1:**
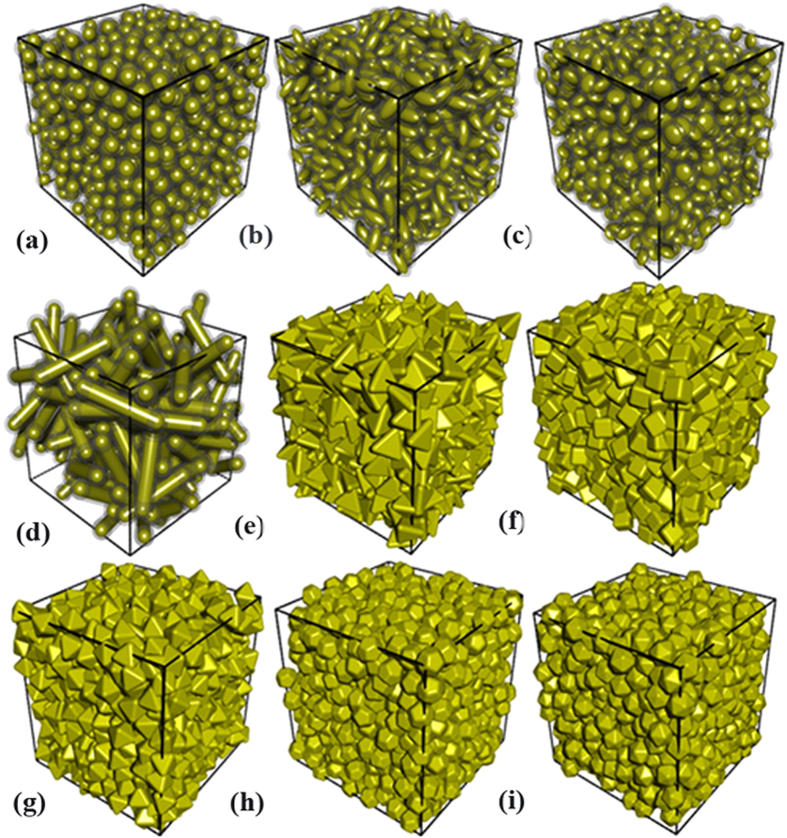
Schematic views of geometric models of composite materials constructed by hard-core-soft-shell structured particles with monodisperse particle systems. (**a**) Spheres, (**b**) prolate ellipsoids (*κ* = 2.5, *κ* is the aspect ratio), (**c**) oblate ellipsoids (*κ* = 0.6), (**d**) spherocylinders (*H*/*D* = 2.5, *H* and *D* are the height and diameter of a sphrocylinder, respectively.), (**e**) tetrahedra, (**f**) hexahedra, (**g**) octahedra, (**h**) dodecahedra, and (**i**) icosahedra. The interfacial dimension *t*, particle volume fraction *V*_*p*_, and cubic cell dimension *L* are set to be *t* = 2.0, *V*_*p*_ = 0.32, and *L* = 100, respectively.

We start our computer simulations by packing non-spherical particles like spheroids, spherocylinders, regular tetrahedrons, hexahedrons, octahedrons, dodecahedrons, and icosahedrons into a matrix viewed as a cubic structure. We create macroscopic assemblages of hard particles using a random sequential addition procedure^26^,^27^. The details of random sequential addition procedure are briefly reviewed in the Supplementary methods (S9). Then, a uniform interfacial shell described above is coated on the surface of each hard particle, as well as the adjacent interfaces may intersect in order to cater for the HCSS structure. As schematic drawings, Fig. 1 depicts nine three-phase composite models composed of matrix, interfaces, and monodisperse non-spherical particles of nine geometric shapes. Fig. S3 shows further packing systems of polydisperse non-spherical particles with a uniform interfacial shell (see Supplementary Information, Fig. S3). Subsequently, a Monte Carlo random point sampling algorithm is performed to obtain the interfacial volume fraction, and its operation strategy is executed in the Supplementary methods (S10).

## Discussion

In this section, some results on the influences of particle geometries on the interfacial volume fraction are displayed and discussed by the proposed theoretical and numerical approaches. We employ monodisperse particle systems to investigate the effects of geometric size factor and particle shape so as to explicitly reveal the mechanisms of geometric features of particles. On the other hand, the impact of particle size distribution is described in Supplementary results (S11).

**Figure 2:**
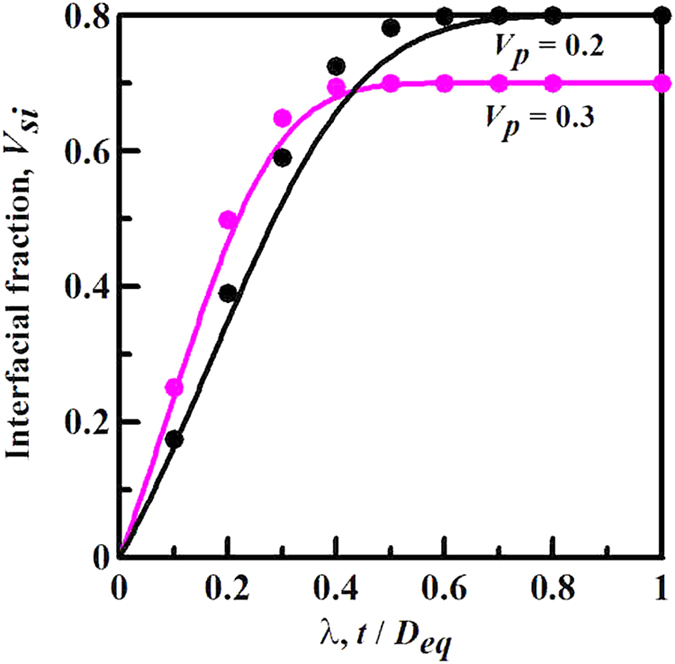
Effect of geometric size factor λ of particles on the interfacial volume fraction V_si_ for two given particle volume fractions of Vp = 0.2 and 0.3. The lines and circular points denote theoretical and numerical results, respectively. Numerical experiments are implemented in monodisperse octahedron (*s* = 0.846) particle systems with the constant interfacial dimension of *t* = 0.03.

Figure 2 displays the effect of geometric size factor *λ* of anisotropic particles on the interfacial volume fraction *V*_*si*_ by theoretical and numerical methods presented above. From Fig. 2, it can be seen that the theoretical results are in good agreement with the numerical results of *V*_*si*_ for different *λ* and *V*_*p*_. It indicates that the stability of the present theoretical scheme for the interfacial volume fraction of monodisperse anisotropic particle systems is favorable. On the other hand, under the same particle volume fraction, *V*_*si*_ firstly increases with the increase of *λ*. When *λ* increases to a critical value, *V*_*si*_ keeps a stable value equal to 1–*V*_*p*_, in other words, the interfaces overlap enough to fill up the remaining matrix as *λ* reaches a threshold. This reflects that the proposed theoretical scheme exactly provides an efficient method for obtaining the threshold. It seems that it is the first time to our knowledge that such a correlation is presented. In addition, according to the definition of geometric size factor of *λ* = *t*/*D*_*eq*_, we can find that the interfacial volume fraction intensively depends on the coupling of interfacial dimension and equivalent diameter, rather than the single factor like interfacial dimension or equivalent diameter. It is an interesting finding that can allow researchers to just optimize the geometric size factor to design the interfacial property in composite materials. To verify the validity of interesting generalization, we further test the effect of single factor on the interfacial volume fraction with a fixed geometric size factor.

**Figure 3:**
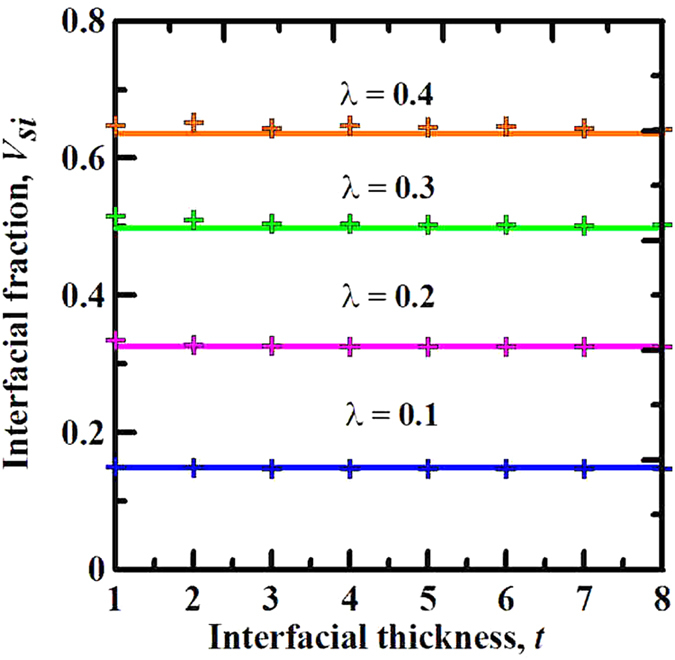
Comparison of the same geometric size factor with different interfacial dimensions. Effect of the interfacial dimension *t* on the interfacial volume fraction *V*_*si*_. The lines and cross-shaped points represent theoretical and numerical results, respectively. Numerical experiments are executed in monodisperse oblate ellipsoid (*s* = 0.562) particle systems with the constant particle volume fraction of *V*_*p*_ = 0.2.

Without the loss of generality, we use the theoretical and numerical models to evaluate the influence of interfacial dimension *t* on the interfacial volume fraction with a constant geometric size factor, in which constant geometric size factors of five kinds are investigated, as shown in Fig. 3. As expected, Fig. 3 shows that, for each fixed *λ*, the theoretical and numerical results of *V*_*si*_ are completely invariants with varying the interfacial dimension. This confirms the validity of the above generalization. To be specific, in a monodisperse particle system, the interfacial volume fraction is only limited by the geometric size factor under the constant particle shape and volume fraction.

**Figure 4:**
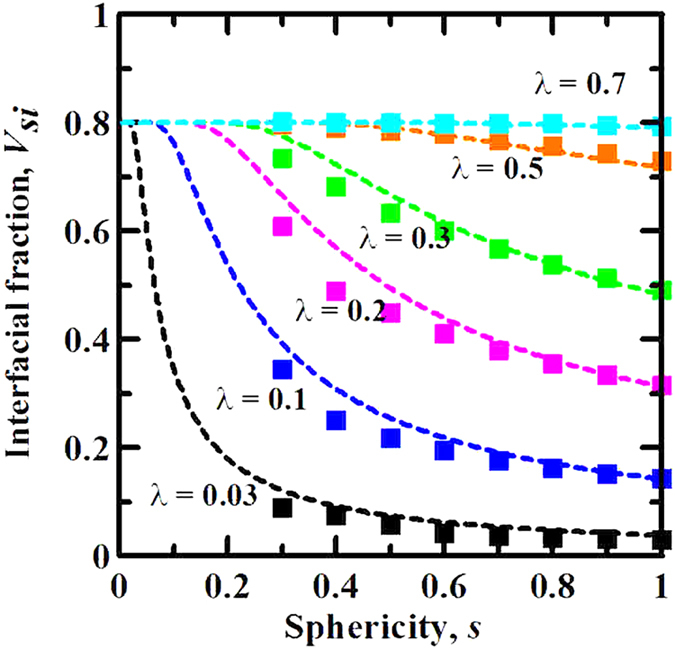
Effect of particle shape characterized by sphericity s on the interfacial volume fraction Vsi in monodisperse particle systems. The dashed lines and cubic-shaped points represent theoretical and numerical results, respectively. Numerical experiments are executed with the constant particle volume fraction of *V*_*p*_ = 0.2 and interfacial dimension of *t* = 5.

Figure 4 depicts the effect of sphericity *s* as the shape descriptor of anisotropic particles on the interfacial volume fraction for various geometric size factors *λ* by theoretical and numerical models. For the smaller *λ*, Fig. 4 shows that *V*_*si*_ decreases with the increase of *s*, and reaches the minimal value with *s* = 1, i.e., spheres. It means that anisotropic particles give rise to an increment of interfacial volume fraction with respect to isotropic spheres. Thereby, the derived results demonstrate the assumption of spherical particles underestimates the interfacial volume fraction in composite materials. The case numerically attributes to the sharp increase of the packing fraction of anisotropic particles as particles deviate from spheres reported in relevant references^17^,^23^,^28^,^29^,^30^. Indeed, the packing of hard particles is denser; the overlap potential between interfaces around particles is more significant, which induces the expected increase of interfacial volume fraction. Experimentally, The reason is that the required amount of matrix for wrapping the surface of spherical particles is lower than that for wrapping the surface of anisotropic particles, so that interfaces generated in the vicinity of spherical particles are less than those generated in the vicinity of anisotropic particles, since the packing constraint of the particle surface on matrix gives rise to the formation of interfaces in some composite materials, such as ceramic and cementitious composites.

Interestingly, for the larger *λ*, e.g., *λ* = 0.7, theoretical and numerical results consistently display that *V*_*si*_ keeps basically the constant (*V*_*si*_ = 1 − *V*_*p*_ = 0.8) with *s*, as shown in Fig. 4. In other words, the volume of interfaces occupies to the whole matrix in composite materials. It reveals that the response of interfacial volume fraction on the geometric size factor is more sensitive than that on the geometric shape of anisotropic particles. It is worth pointing out that the present theoretical results just take into account sphericity of particles regardless of different types of anisotropic particles, but the numerical results reported in Fig. 4 originate from spheroids of different aspect ratios, according to the relation between aspect ratio and sphericity, that is


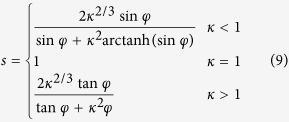


where *κ* is the aspect ratio of a spheroid, *φ* is defined as *φ* = arcos (*η*), *η* = 1/*κ* (*κ* > 1) or *η* = *κ* (*κ* < 1). Although the numerical results are in good line with the theoretical results for spheroids, as shown in Fig. 4, it is unclear whether the present theoretical framework is suitable for other anisotropic particles with the same sphericity. So, it is very necessary to address the gap in the following.

**Figure 5:**
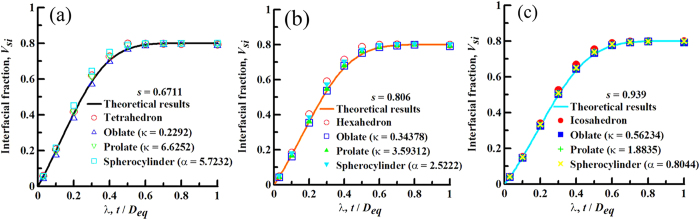
Comparisons of Vsi of the same s with different particle types. Three groups of the numerical experiments are set as **(a)** *s* = 0.6711, **(b)** *s* = 0.806, and **(c)** *s* = 0.939. Each group contains four types of anisotropic particles of the same sphericity. The lines and points represent theoretical and numerical results, respectively. Note that basic parameters in Fig. 5 are in agreement with those in Fig. 4.

In order to discuss the issue, we set numerical experiments of three groups, and each group possessing a uniform sphericity contains four types of anisotropic particles, such as convex polyhedrons, prolate spheroids, oblate spheroids, and spherocylinders, as shown in Fig. 5. With the present numerical model applied, the numerical results of interfacial volume fraction for anisotropic particles of different types are derived. We compare the numerical experimental results of each group with the theoretical solution of interfacial volume fraction for a constant sphericity, as shown in Fig. 5. For the first group experiment with the uniform *s* = 0.6711, Fig. 5(a) presents the numerical results for tetrahedrons, oblate spheroids of *κ* = 0.2292, prolate spheroids of *κ* *=* 6.6252, and spherocylinders of the aspect ratio of *α* = 5.7232 are so in good agreement with the theoretical solution of interfacial volume fraction for various geometric size factors, even if the numerical results for four types of anisotropic particles are slightly distinguishable each other, because of the inevitable machine errors. It is noted that *α* is the aspect ratio of a spherocylinder, i.e., *α* = *H*/*D*, In terms of the definition of sphericity, the relation between *α* and *s* is expressed as


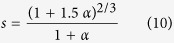


Moreover, for the other two groups, Fig. 5(b,c) illustrate the same rule in relation to Fig. 5(a). Therefore, the proposed theoretical framework as a generalized scheme integrates the interfacial volume fraction for anisotropic hard particles of different geometric types. Also, it is required that we need only to input the geometric shape factor, i.e., sphericity *s*, of anisotropic particles for determining the interfacial volume fraction under the same other parameters, no longer need a case-by-case consideration.

## Methods

As a theoretical foundation of the interfacial volume fraction, the theory of the nearest-surface distribution functions will be reviewed briefly to formulate the interfacial volume fraction. In the theory, a so-called void “exclusion” probability of composite media composed of spherical particles and voids is derived by the statistical geometry of composites and geometric probability^12^


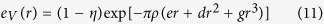


with


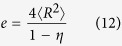



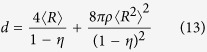



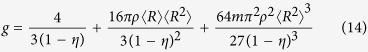


where *e*_*V*_ (*r*) is defined as the probability of finding a region that is a spherical cavity of radius *r* centered at some arbitrary point, empty of such composite media. In practice, the void exclusion probability *e*_*V*_ (*r*) displays the expected fraction of voids in such two-phase composites^10^. *ρ* is the number density of spherical particles with radius *R*, and *η* is a dimensionless reduced density defined by


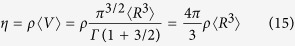


where 〈*V*〉 is the number-averaged volume of hard particles, Г (*x*) is the gamma function. From equation (15) denotes actually the volume fraction of hard spherical particles in three-dimensional space.

Suppose that a composite material consists of hard spherical particles and matrix, *e*_*V*_ (*r*) described above can thus be mapped into the volume fraction of matrix in a two-phase composite material. Similarly, if considering a three-phase composite material of HCSS structure, namely, mono-/polydisperse hard particles of complex geometries, interfaces with a constant dimension of *t* = *r* coating around hard particles, and matrix, as shown in Fig. 1 and Fig. S3. The quantity *e*_*V*_ (*t*) can still characterize the volume fraction of matrix besides hard cores and soft shells, since each particle and its interface can be viewed as a composite particle, so that the three-phase structure is reduced to a two-phase system containing composite particles and matrix.

Nevertheless, for such three-phase composite materials, *e*_*V*_ (*t*) does not follow equations (11)–(14), 12, 13, 14, but it is subjected to geometric configurations of anisotropic particles. To identify geometric details of particles, we rearrange equations (11)–(14), 12, 13, 14:


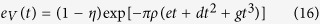


with


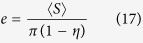



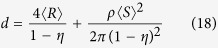



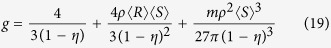


where 〈*S*〉 is the number-averaged surface area of hard particles. According to equations (16)–(19), 17, 18, 19, we can see that, for spherical particle systems, *e*_*V*_ (*t*) mainly relies on the size (*R*), number density (*ρ*), and surface area (*S*) of spherical particles. Likewise, for anisotropic particle systems, *e*_*V*_ (*t*) should be controlled by those geometric characteristics of anisotropic particles. Hence, the key component is how to determine these characteristic parameters of complex geometric particle systems.

In accordance with the preponderance of previous researches, an equivalent diameter *D*_*eq*_ has been broadly employed to represent the size of a non-spherical particle^14^,^19^. Herein, *D*_*eq*_ is still adopted as the size of a complex geometric particle. The number density of anisotropic particle systems can be unbiasedly estimated by quantitative stereological theory^31^, which equals to the ratio of the volume fraction of solid phase to the average volume of hard particles, or the ratio of the specific surface area of solid phase to the average surface area of hard particles. In terms of the definition of *D*_*eq*_, the average volume 〈*V*〉 and surface area 〈*S*〉 of hard non-spherical particles can be given by


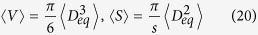


The volume fraction of solid phase with various geometries has been confirmed equivalent approximately to the volume fraction of hard particles by numerical experiments^26^,^32^. Thus, the number density *N*_*V*_ of anisotropic particle systems is given by the stereological theory


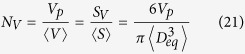


where *S*_*V*_ is the specific surface area of hard anisotropic particles. In the submission of equations (20) and (21) into equations (16)–(19), 17, 18, 19, *e*_*V*_ (*t*) for HCSS structures with complex geometries can be expressed by


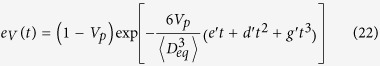


where *e*′, *t*′, and *g*′ are parameters written as equations (2), (3), and (4), respectively. Consequently, for three-phase composite materials, the interfacial volume fraction is the remainder outside all the hard particles and matrix, that is


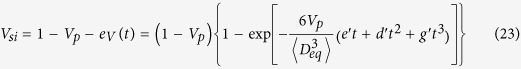


As an application, we also present the theoretical framework how to apply to estimate the effective diffusivity of composite materials, which is demonstrated in the Supplementary results (S12).

## Additional Information

**How to cite this article**: Xu, W. *et al.* Interfacial effect on physical properties of composite media: Interfacial volume fraction with non-spherical hard-core-soft-shell-structured particles. *Sci. Rep.* **5**, 16003; doi: 10.1038/srep16003 (2015).

## Supplementary Material

Supplementary Information

## References

[b1] Sagis L. M. C. Dynamic properties of interfaces in soft matter: Experiments and theory. Rev. Mod. Phys. 83, 1367–1403 (2011).

[b2] Lan X. Z., Masala S. & Sargent E. H. Charge-extraction strategies for colloidal quantum dot photovoltaics. Nat. Mater. 13, 233–240 (2014). 24553652 10.1038/nmat3816

[b3] Estrader M. *et al.* Robust antiferromagnetic coupling in hard-soft bi-magnetic core/shell nanoparticles. Nat. Commun. 4, 2960 (2013) 24343382 10.1038/ncomms3960

[b4] Torquato S. Bulk properties of two-phase media. I. cluster expansion for the dielectric constant of dispersions of fully penetrable spheres. J. Chem. Phys. 81, 5079–5088 (1984).

[b5] Shen Y., Lin Y. H. & Nan C. W. Interfacial effect on dielectric properties of polymer nanocomposites filled with core/shell-structured particles. Adv. Funct. Mater. 17, 2405–2410 (2007).

[b6] Shen Y., Lin Y. H., Li M. & Nan C. W. High dielectric performance of polymer composite films induced by a percolating interparticle barrier layer. Adv. Mater. 19, 1418–1422 (2007).

[b7] Gao Y., Schutter G. D. & Ye G. Micro- and meso-scale pore structure in mortar in realtion to aggregate content. Cem. Concr. Res. 52, 149–160 (2013).

[b8] Xu W. X., Chen H. S., Chen W. & Jiang L. H. Prediction of transport behaviors of particulate composites considering microstructures of soft interfacial layers around ellipsoidal aggregate particles. Soft Matter 10, 627–638 (2014). 24651952 10.1039/c3sm52718b

[b9] Hashin Z. & Shtrikman S. A variational approach to the theory of the effective magnetic permeability of multiphase materials. J. Appl. Phys. 33, 3125–3131 (1962).

[b10] Torquato S. Ra′ndom Heterogeneous Materials: Microstructure and Macroscopic Properties (Springer, 2002).

[b11] Wang M. & Pan N. Predictions of effective physical properties of complex multiphase materials. Mater. Sci. Eng. R 63, 1–30 (2008).

[b12] Lu B. L. & Torquato S. Nearest-surface distribution functions for polydispersed particle systems. Phys. Rev. A 45, 5530–5544 (1992). 9907651 10.1103/physreva.45.5530

[b13] Garboczi E. J. & Bentz D. P. Analytical formulas for interfacial transition zone properties. Adv. Cem. Based Mater. 6, 99–108 (1997).

[b14] Xu W. X. & Chen H. S. Analytical and modeling investigations of volume fraction of interfacial layers around ellipsoidal aggregate particles in multiphase materials. Model. Simul. Mater. Sci. Eng. 21, 015005 (2013).

[b15] Zheng J. J., Guo Z. Q., Pan X. D., Stroeven P. & Sluys L. J. ITZ volume fraction in concrete with spheroidal aggregate particles and application: part I. Numerical algorithm. Mag. Concr. Res. 63, 473–482 (2012).

[b16] Agarwal U. & Escobedo F. A. Mesophase behavior of polyhedral particles. Nat. Mater. 10, 230–235 (2011). 21317901 10.1038/nmat2959

[b17] Baule A., Mari R., Bo L., Portal L. & Makse H. A. Mean-field theory of random close packings of axisymmetric particles. Nat. Commun. 4, 2194 (2013). 23877324 10.1038/ncomms3194

[b18] Wei Z.-Y. & Matsui H. Rational strategy for shaped nanomaterial synthesis in reverse micelle reactor. Nat. Commun. 5, 3870 (2014). 24828960 10.1038/ncomms4870 PMC4112590

[b19] Xu W. X., Chen W. & Chen H. S. Modeling of soft interfacial volume fraction in composite materials with complex convex particles. J. Chem. Phys. 140, 034704 (2014). 25669404 10.1063/1.4861664

[b20] Lebowitz J. L. Exact solution of generalized Percus-Yevick equation for a mixture of hard spheres. Phys. Rev. 133, A895–A899 (1964).

[b21] Mansoori G. A., Carnahan N. F., Starling K. E. & Leland T. W. Jr Equilibrium thermodynamic properties of the mixture of hard spheres. J. Chem. Phys. 54, 1523–1525 (1971).

[b22] Lebowitz J. L., Helfand E. & Praesegaad E. Scaled particle theory of fluid mixtures. J. Chem. Phys. 43, 774–779 (1965).

[b23] Zhou Z. Y., Zou R. P., Pinson D. & Yu A. B. Dynamic simulation of the packing of ellipsoidal particles. Ind. Eng. Chem. Res. 50, 9787–9798 (2011).

[b24] Boton M., Azema E., Estrada N., Radjai F. & Lizcano A. Quasistatic rheology and microstructural description of sheared granular materials composed of platy particles. Phys. Rev. E 87, 032206 (2013).

[b25] Xu W. X., Chen H. S., Duan Q. L. & Chen W. Strategy for interfacial overlapping degree in multiphase materials with complex convex particles. Powder Technol. 283, 455–461 (2015).

[b26] Xu W. X. & Chen H. S. Numerical investigation of effect of particle shape and particle size distribution on fresh cement paste microstructure via random sequential packing of dodecahedral cement particles. Comput. Struct. 114-115, 35–45 (2013).

[b27] Zhang G. & Torquato S. Precise algorithm to generate random sequential addition of hard hyperspheres at saturation. Phys. Rev. E 88, 053312 (2013). 10.1103/PhysRevE.88.053312 24329384

[b28] Zhao J., Li S. X., Zou R. P. & Yu A. B. Dense random packings of spherocylinders. Soft Matter 8, 1003–1009 (2012).

[b29] Torquato S. & Jiao Y. Dense packings of the Platonic and Archimedean solids. Nature 460, 876–879 (2009). 19675649 10.1038/nature08239

[b30] Donev A. *et al.* Improving the density of jammed disordered packings using ellipsoids. Science 303, 990–993 (2004). 14963324 10.1126/science.1093010

[b31] Underwood E. E. Quantitative Stereology (Addison-Wesley, 1968).

[b32] Yang R. Y., Zou R. P. & Yu A. B. Computer simulation of the packing of fine particles. Phys. Rev. E 62, 3900–3908 (2000). 10.1103/physreve.62.3900 11088910

