# Supplementary material for: Interfacial effect on physical properties of composite media: Interfacial volume fraction with non-spherical hard-core-soft-shell-structured particles
Source: Sci Rep. 2015 Nov 2;5:16003. doi: 10.1038/srep16003 (PMC4629176; doi:10.1038/srep16003)
Supplement: Supplementary Information [file srep16003-s1.doc]

**Interfacial effect on physical properties of composite media: Interfacial volume fraction with non-spherical hard-core-soft-shell-structured particles**

Wenxiang Xu[[1]](#footnote-2) 1, 2, 3, Qinglin Duan^2^, Huaifa Ma^3^, Wen Chen^1^ and Huisu Chen^4^


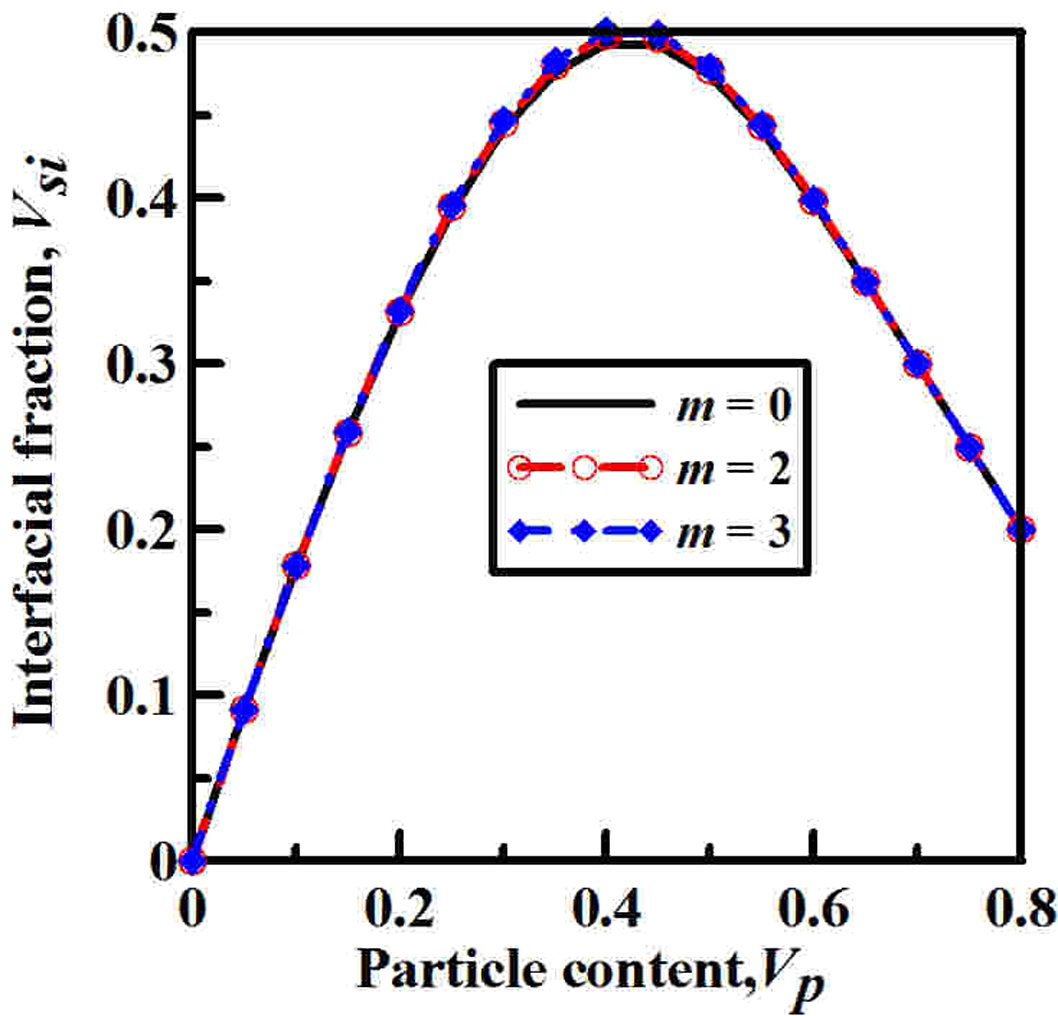


**Supplementary Figure S1.** **The effect of parameter *m* on the interfacial volume fraction *V_si_***. Effect of the parameter *m* on the theoretical results of interfacial volume fraction *V_si_* around monodisperse anisotropic particles for various particle contents *V_p_*. Theoretical results of *V_si_* with *t* = 0.03, *D_eq_* = 0.15, and *s* = 0.91.

**
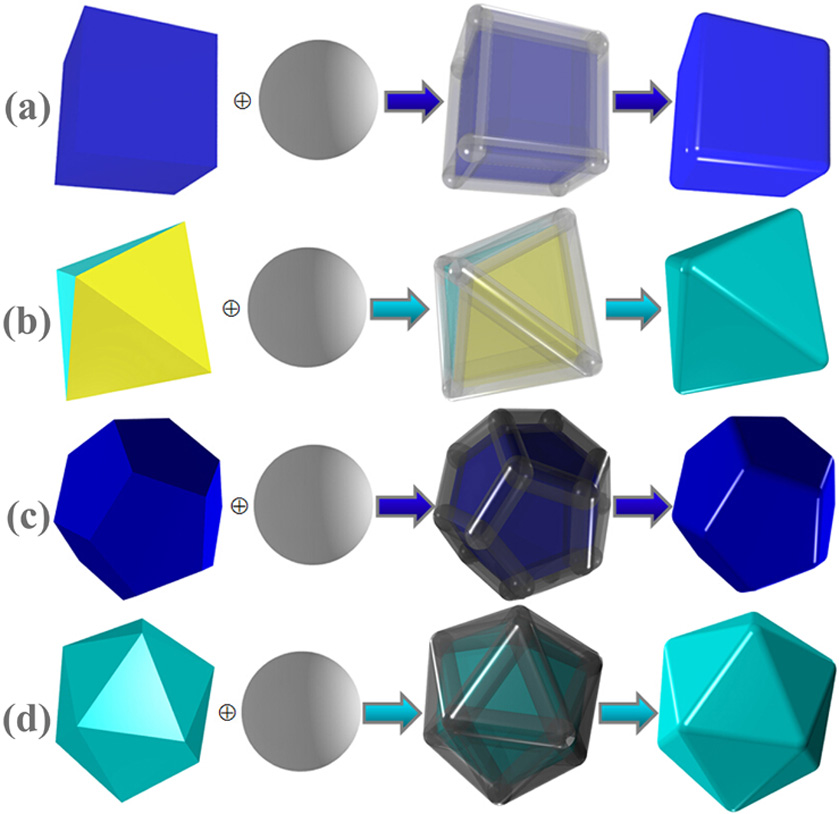
**

**Supplementary Figure S2.** **Geometric realizations by the Minkowski addition manner.** The realistic geometric configuration of interfacial shell with a constant dimension around a non-spherical particle like **(a)** hexahedron, **(b)** octahedron, **(c)** dodecahedron, and **(d)** icosahedron.

**
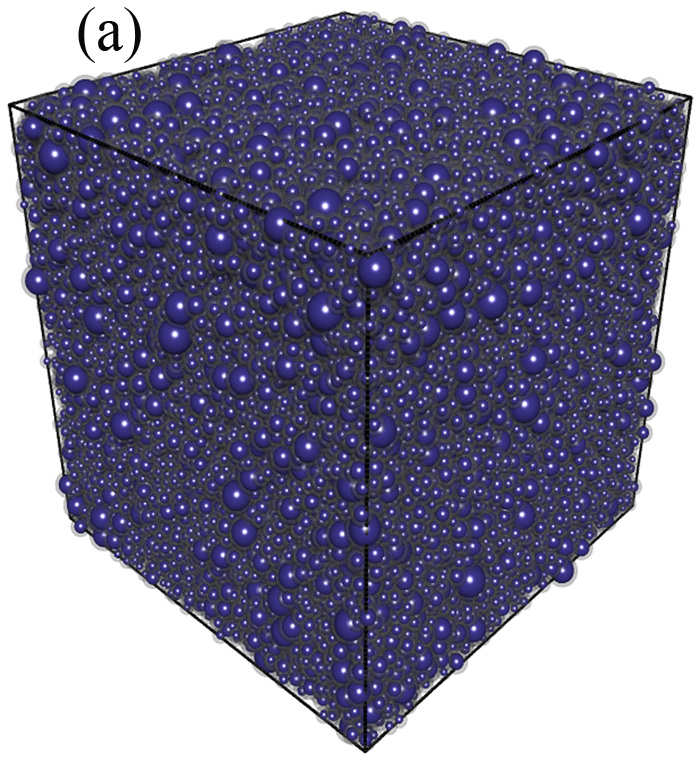

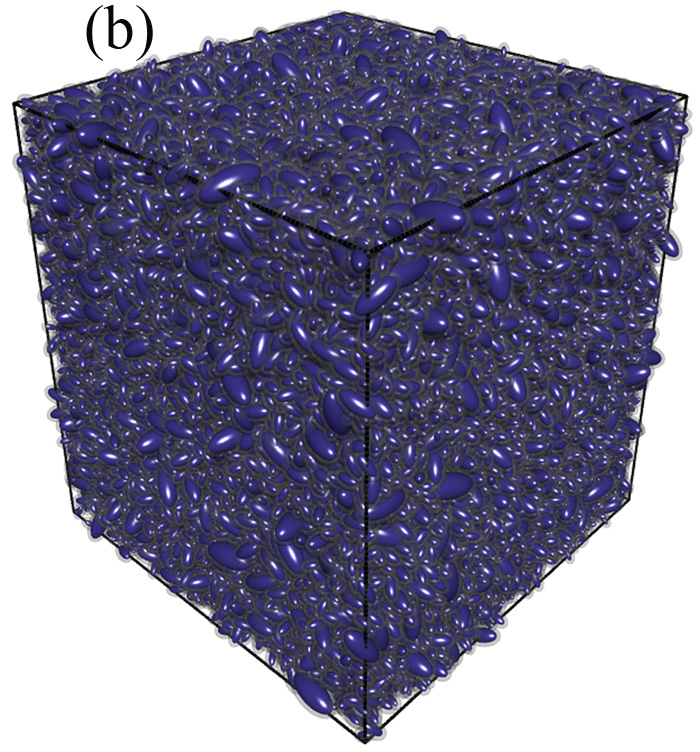

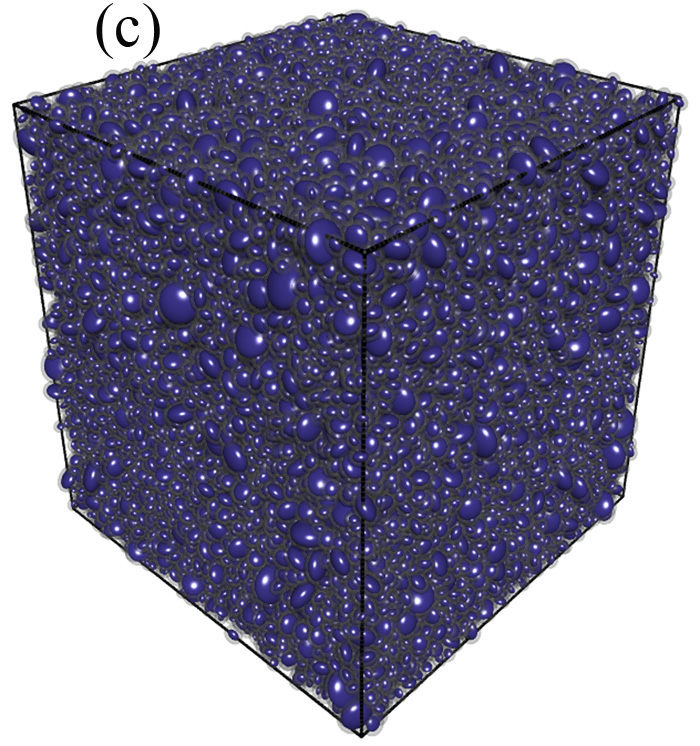
**

**
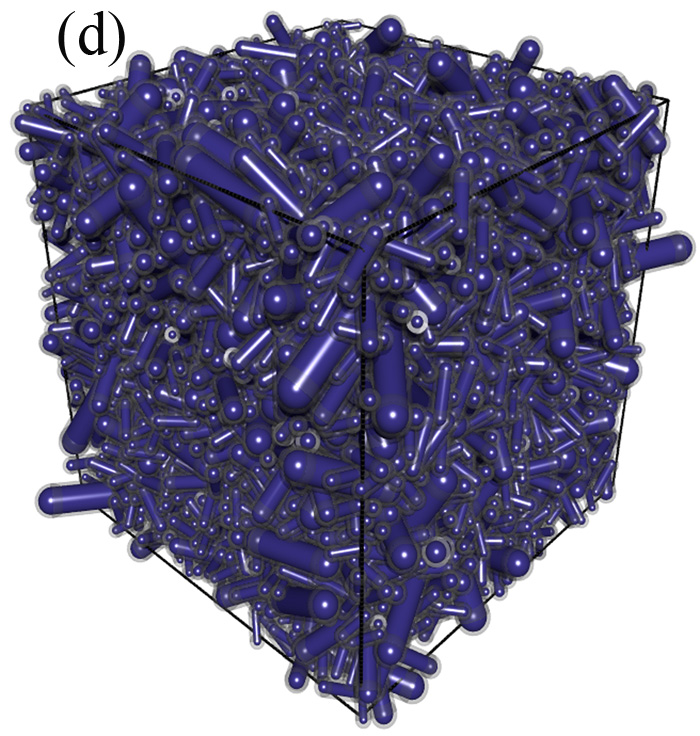

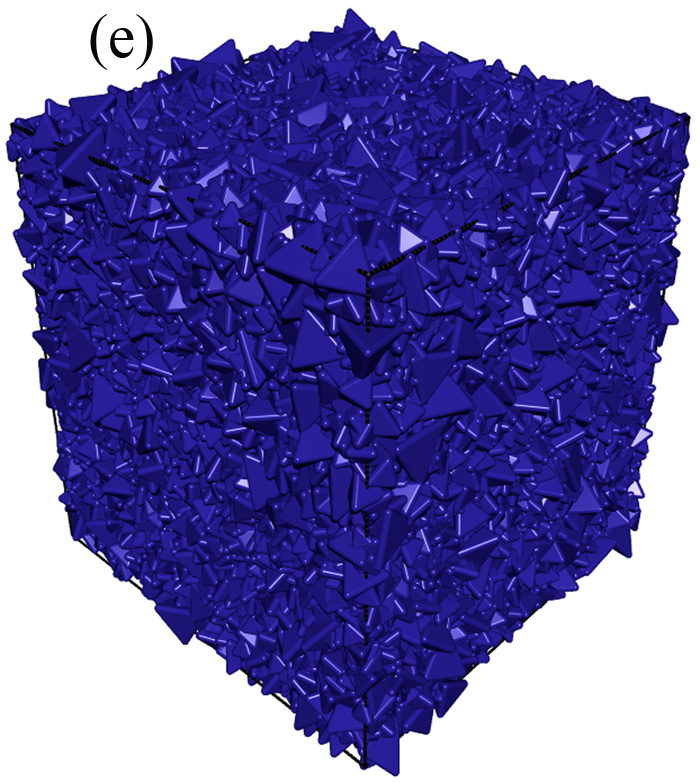

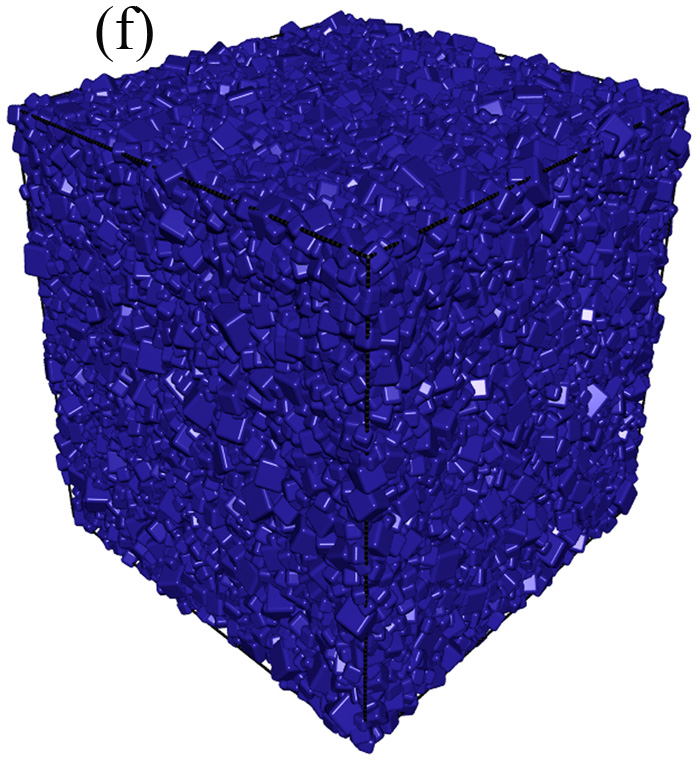
**

**
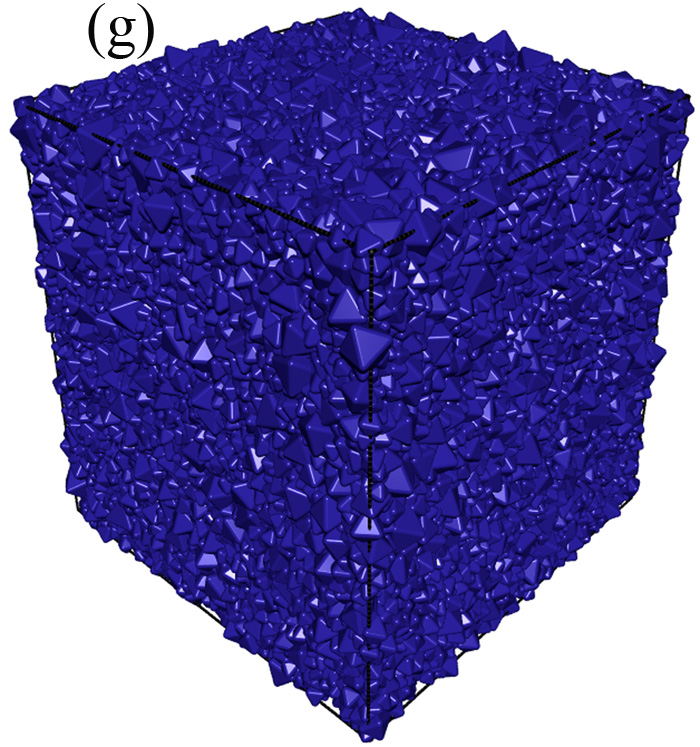

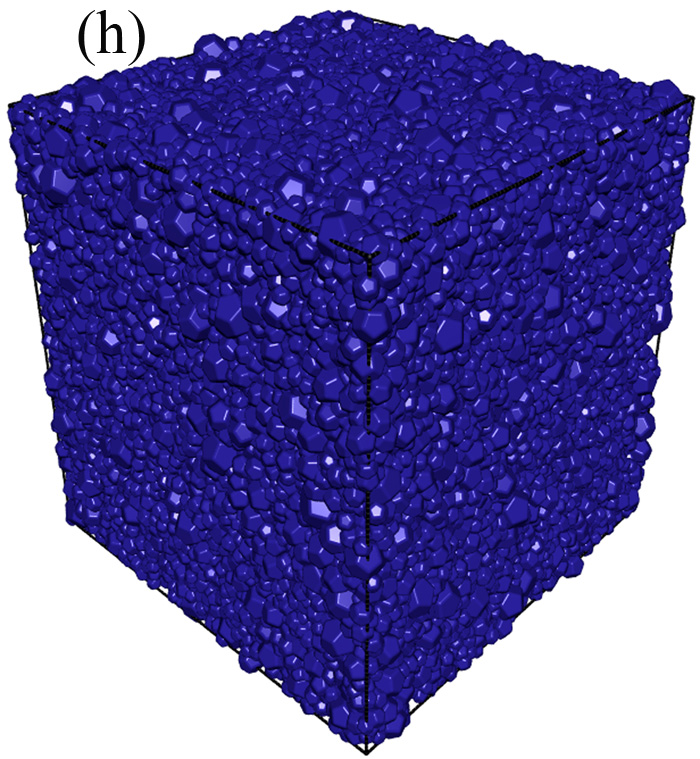

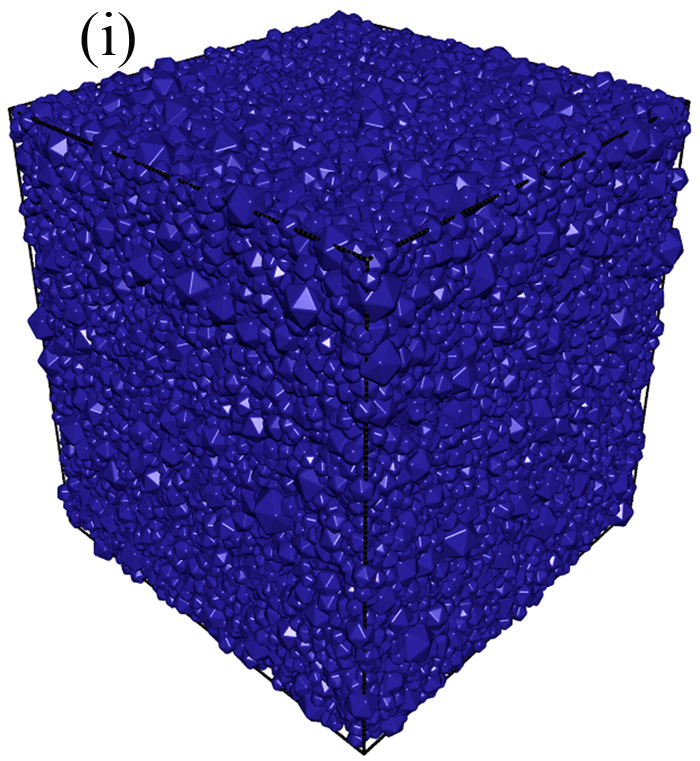
**

**Supplementary Figure S3.** **Three-phase composite structures consisting of soft interfaces of a constant dimension, matrix, and polydisperse particles.** **(a)** spheres, **(b)** prolate ellipsoids (*κ* = 2.5), **(c)** oblate ellipsoids (*κ* = 0.6), **(d)** spherocylinders (*H*/*D* = 3.5), **(e)** tetrahedra, **(f)** hexahedra, **(g)** octahedra, **(h)** dodecahedra, and **(i)** icosahedra. The particle size distribution is assigned as the equal volume fraction distribution, the interfacial dimension *t*, the maximum and minimum equivalent diameters *D*_max_*_eq_* and *D*_min_*_eq_*, the particle volume fraction *V_p_*, and cubic cell dimension *L* are set to be *t* = 2.5, *D*_max_*_eq_* = 20, *D*_min_*_eq_* = 5, *V_p_* = 0.5, and *L* = 300, respectively.

**
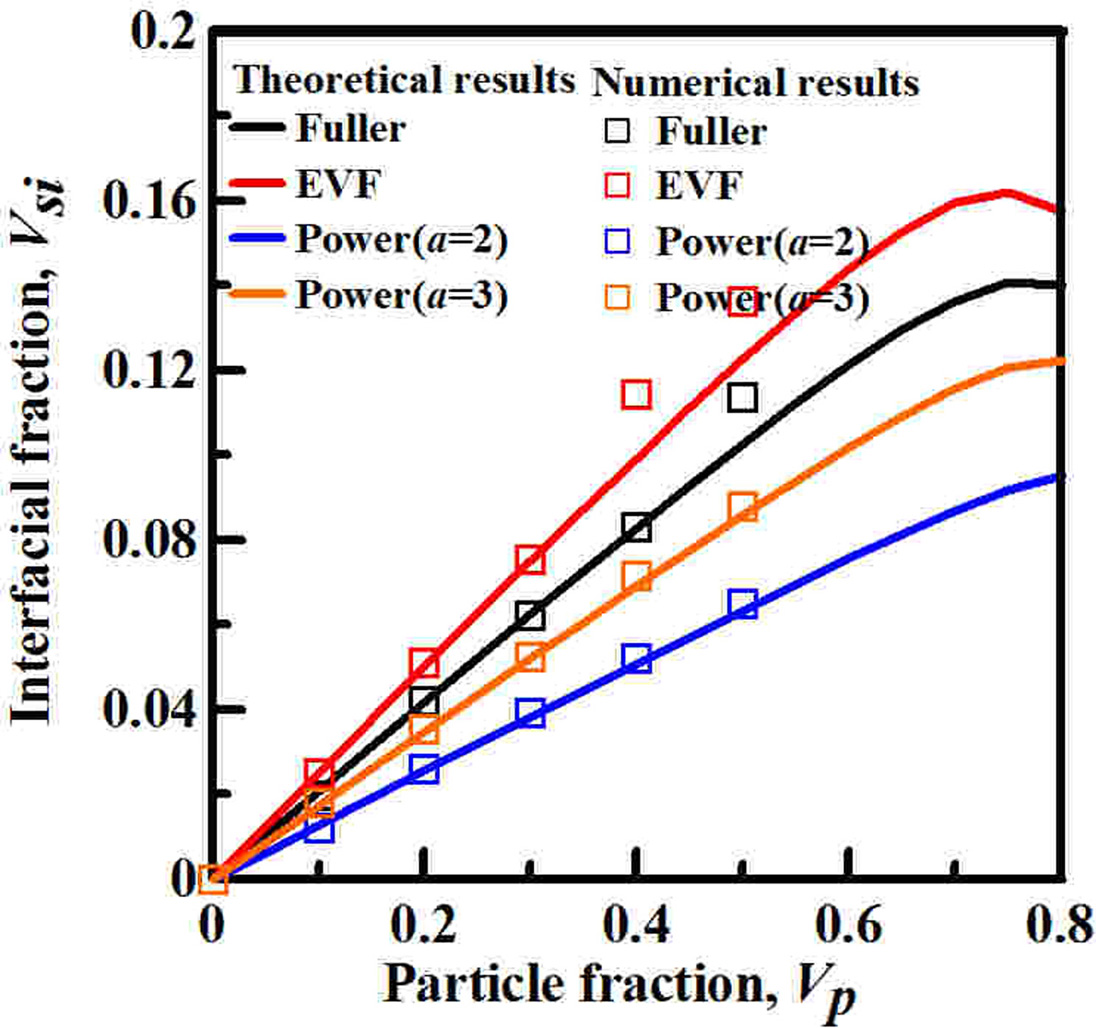
**

**Supplementary Figure S4.** **The interfacial volume fraction *V_si_* versus particle size distribution for different particle volume fractions *V_p_*.** The other basic parameters are set to be *D*_min_*_eq_* = 5.0, *D*_max_*_eq_* = 40.0, and *t* = 0.3, Numerical experiments are executed in polydisperse spherocylinder particle systems with *α* = 1.116 (*s* = 0.9104). The cubic points and lines depict the numerical and theoretical results, respectively.

**
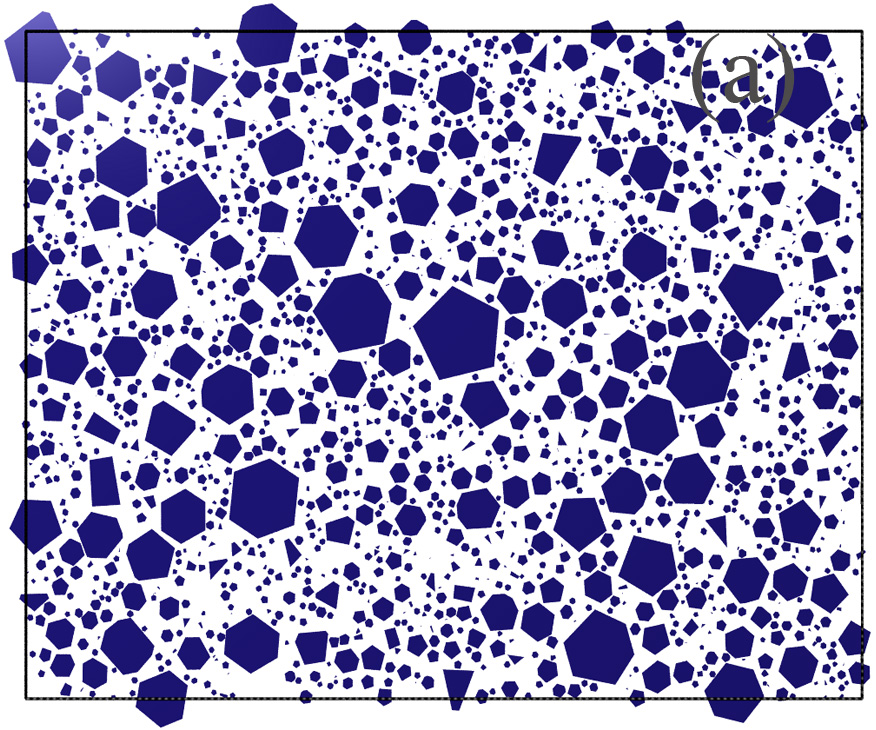

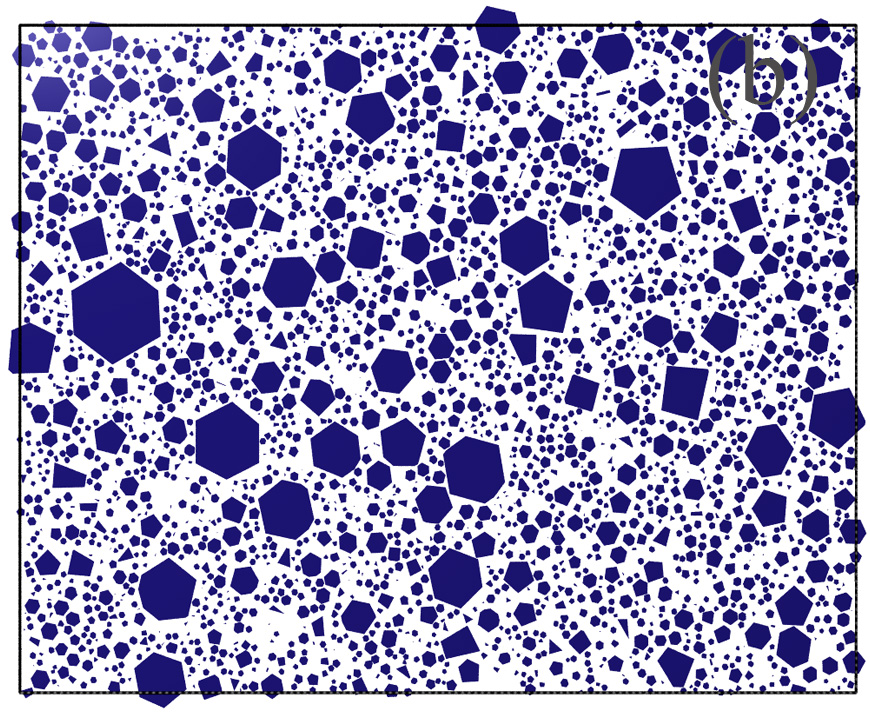
**

**Supplementary Figure S5.** **Visualizations of two-dimensional cross-sections**. Two-dimensional cross-section configurations of polydisperse dodecahedral particle systems with the power-law distribution of the exponent: **(a)** *a* = 3.5 and **(b)** *a* = 4. *V_p_* = 0.43.

**
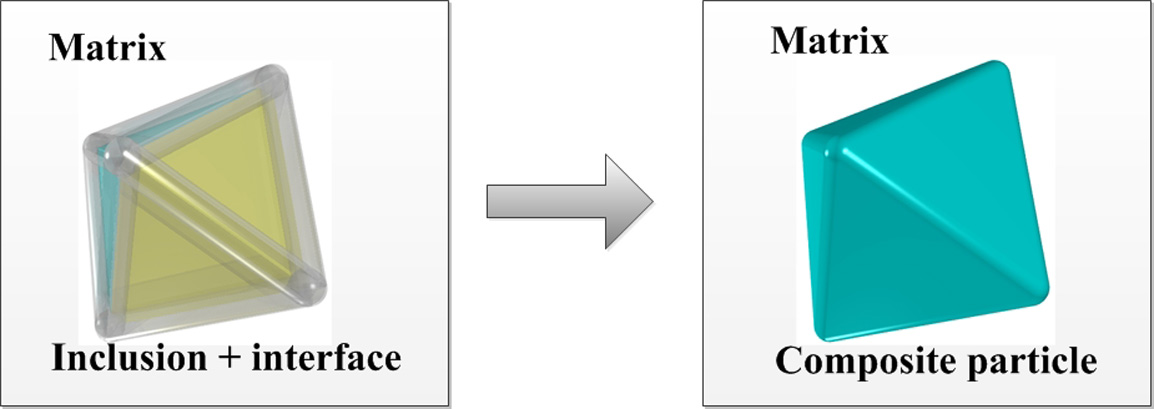
**

**Supplementary Figure S6.** **Schematic views for mapping of a three-phase composite structure**. The three-phase composite structure with hard inclusion, interface, and matrix into a two-phase structure composed of the composite particle and matrix.

**
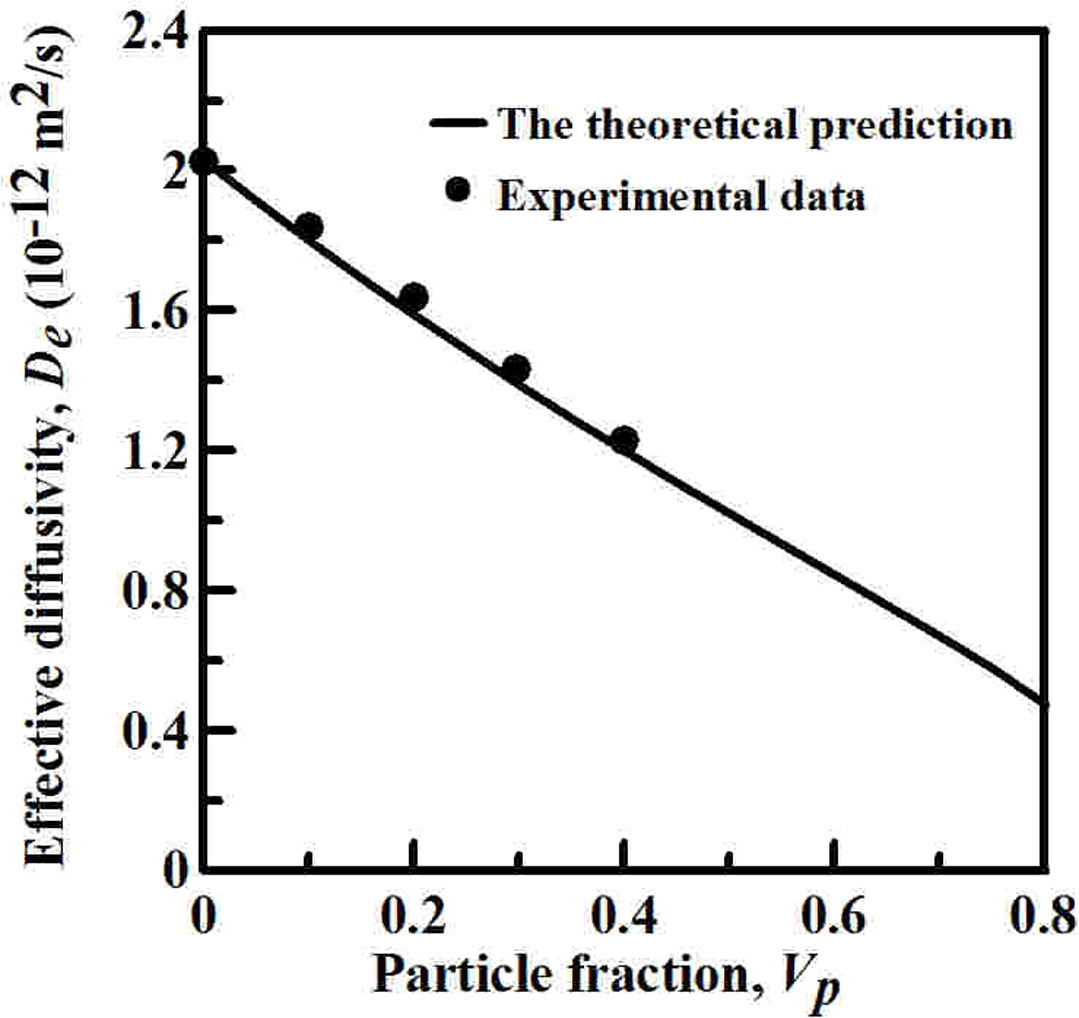
**

**Supplementary Figure S7.** **Comparisons of the theoretical prediction results of effective diffusivity of composite materials containing interfaces with the experimental data of diffusivity from the accelerate chloride ion migration experiment**. Basic parameters of the theoretical prediction refer to experimental test^18,19^, such as the interfacial dimension *t* = 0.04, particle size range of *D*_min_*_eq_* = 0.15 and *D*_max_*_eq_* = 9.5, Fuller gradation, and particle shape is characterized by the aspect ratio of *κ* = 2.021from X-ray CT scanner experiments^19^.

**Supplementary methods**

**S8: The interfacial volume fraction for polydisperse anisotropic particle systems**

The investigation of polydisperse particle systems is to account for the impact of particle size distribution (PSD) on the interfacial volume fraction. However, PSD of anisotropic particles is seldom referred in theoretical and numerical studies. Experimentally, a gradation of irregular aggregates is usually measured by the conventional sieve analysis or laser particle analyzer, where the size of each irregular aggregate is macroscopically analyzed relative to circular screen or spherical particle size^1^. Herein, we follow the preliminary studies^2,3^ that adopt the experimental principle to the theoretical and numerical procedures. To be specific, PSD of spherical particles is transformed to characterize PSD of anisotropic particles by the definition of equivalent diameter. We example three particular gradations, such as the power-law^4^, Fuller (that essentially is a particular power-law distribution function with the exponent of *a*=3.5, which is normally used to represent the aggregate gradation in cementitous and ceramic materials^1-3^), and equal volume fraction (EVF) distribution functions^1-3^, which reflect ordinarily spherical aggregate size distribution in particulate composite materials:

$f_{N}\left(R\right)=\frac{-q}{2R^{q+1}\left(R_{\max}^{-q}-R_{\min}^{-q}\right)}\text{ }\left\{\begin{matrix} q=2.5\to\text{Fuller} \\ q=3.0\to\text{EVF} \end{matrix}\right.$ (1)

$f_{N}\left(R\right)=\eta R^{-a}\text{ }\left\{\begin{matrix} \eta=\frac{a-1}{R_{\min}^{1-a}-R_{\max}^{1-a}}\to a\neq1 \\ \eta=\left(\ln R_{\max}-\ln R_{\min}\right)^{-1}\to a=1 \end{matrix}\right.$ (2)

where *f_N_* (*R*) is the number-based probability function of spherical particle systems, *R*_max_ and *R*_min_ are the maximum and minimum radii of spherical particles, respectively. *a* is an exponent with respect to the power-law distribution, the value of which is usually prescribed in the interval between 1.5 and 4.5^4^. In terms of the definition of *D_eq_*, a PSD of anisotropic particles can be connected with that of equivalent spheres. Consequently, by substituting *D_eq_* into *R* mentioned in the above formulas, the three specific gradations for anisotropic particles are displayed by

$f_{N}\left(D_{eq}\right)=\frac{-q}{D_{eq}^{q+1}\left(D_{\max eq}^{-q}-D_{\min eq}^{-q}\right)}\text{ }\left\{\begin{matrix} q=2.5\to\text{Fuller} \\ q=3.0\to\text{EVF} \end{matrix}\right.$ (3)

$f_{N}\left(D_{eq}\right)=\eta D_{eq}^{-a}\text{ }\left\{\begin{matrix} \eta=\frac{a-1}{D_{\min eq}^{1-a}-D_{\max eq}^{1-a}}\to a\neq1 \\ \eta=\left(\ln D_{\max eq}-\ln D_{\min eq}\right)^{-1}\to a=1 \end{matrix}\right.$ (4)

where *f_N_* (*D_eq_*) is the number-based probability function of anisotropic particle systems, *D*_max_*_eq_* and *D*_min_*_eq_* are the maximum and minimum equivalent diameters of anisotropic particles. Thus, the *k*th moment 〈*D_eq_^k^*〉 is given by

$\left\langle D_{eq}^{k}\right\rangle=\int_{D_{\min eq}}^{D_{\max eq}} D_{eq}^{k}f_{N}\left(D_{eq}\right)\text{d}D_{eq}$ (5)

In the substitution of equations (3) and (4) into equation (5), 〈*D_eq_*〉, 〈*D_eq_*^2^〉, and 〈*D_eq_*^3^〉 are just determined to obtain the interfacial volume fraction of polydisperse anisotropic particle structures. **S9: Random sequential addition procedure for packing of hard particles**

1. Input control parameters. The key parameters include a cubic cell size *L*, hard particle type (monodisperse or polydisperse) and shape, PSD, and the particle volume fraction *V_p_*. For a polydisperse particle system, the maximum and minimum equivalent diameter of particles *D*_max_*_eq_* and *D*_min_*_eq_* are specified. Alternatively, the equivalent diameter of particles *D_eq_* in a monodisperse particle system is specified.
2. The cubic cell is prescribed periodic boundary conditions to eliminate the wall effect. It means that particles are allowed to intersect with boundary walls, as well as a so-called periodic compensation technology is implemented once a particle intersects with boundary walls^3^.
3. The cell size *L* is assigned to be 20 times to the maximum equivalent diameter of particles to satisfying with the requirement of a representative volume element of structures.
4. In the fixed cubic cell, if considering a polydisperse particle system with a predefined PSD, the number of particles of various sizes related to the particle volume fraction is obtained by a Monte Carlo integration scheme demonstrated in the previous research^3^. For a monodisperse particle system, the number of particles is directly calculated by the given particle size and volume fraction.
5. In a polydisperse particle system, the size of particles is arranged in a descending order in order to improve the packing density of particles.
6. Randomly generate the centroid **C***_i_* of the particle *i* in the cubic cell.
7. Randomly generate three Euler angles *α_i_*, *γ_i_*, and *β_i_* of the particle *i* in the intervals [0, 2π] and [0, π].
8. Judge whether the generated particle *i* intersects with the periodic boundary planes. If intersection, the particle *i* is (virtually) replicated onto corresponding locations by virtue of the periodic compensation technology^3^.
9. Detect whether the generated particle *i* and its duplicate particles (if any) overlap with all those preceding *i*-1 particles or not, if overlapping, return to Steps (6) and (7) to regenerate the particle *i*. Otherwise, return to Steps (6) and (7) to generate the particle *i*+1.
10. Iterate Step (4) to Step (9), until all particles are generated.

It should be mentioned that the key issue of operating the procedure is to identify the inter-particle contact. The overlapping detection between ellipsoidal particles can take advantage of a golden section search numerical algorithm developed in our preliminary studies^5,6^. Xu and co-workers^7^ have recently presented a separation axis scheme to judge the contact between convex polyhedral particles. The overlapping measurement between spherocylinders is referred to the early work introduced by Williams and Philipse^8^. More details on these contact detection algorithms can be found in the literature.

**S10: Monte Carlo random point sampling algorithm**

A lot of random points are generated in three-phase composite structures, and the number of random points is required to meet the reliability and efficiency of numerical algorithm. We adopt the coefficient of variation of the average statistics of interfacial volume fraction as a criterion for determining the number of random points. Herein, the coefficient of variation is set to 0.01. Then, the number of valid random points falling within interfacial shells is recorded. The interfacial volume fraction is thus equivalent to the ratio of the number of valid random points to the total amount of points. In doing so, it is very crucial to identify the valid random points that fall inside the interfacial shells rather than within the original particles. Accordingly, the relative spatial position between a random point and the interfacial shell should be detected. As shown in Fig. S2, the geometric morphology of an interfacial shell originates from a sphere with its radius equivalent to the interfacial dimension sweeping around the original particle. The check of the relative spatial position between a random point and an interfacial shell can thus be transformed to detect the relative spatial position between a point and hemispheres consisting of the rolling trajectory of the sweeping sphere, for which the operation is very simple.

**Supplementary results**

**S11: Effect of PSD on the interfacial volume fraction**

We assess the effect of PSD on the interfacial volume fraction in polydisperse anisotropic particle systems by the present theoretical and numerical models. Fig. S4 displays the dependence of interfacial volume fraction *V_si_* on PSD including Fuller, equal volume fraction (EVF), and power-law with exponents of *a* = 2 and 3 distribution functions, for various particle volume fractions *V_p_*. It can be clearly seen from Fig. S4 that, in polydisperse particle systems with different PSD and *V_p_*, the theoretical results of interfacial volume fraction remain a favorable consistency with that of the numerical results. It further indicates that the proposed theoretical scheme is not only a generalized model for monodisperse anisotropic particle structures, but also suitable for polydisperse anisotropic particle systems.

Besides, from Fig. S4, it can be seen that, for a given PSD, the interfacial volume fraction increases monotonically with the increase of the volume fraction of particles. The higher packing density of hard particles generates the larger interfacial volume fraction. Indeed, the packing of particles is denser; the overlap potential of interfaces around particles is more dramatic^8-10^, which leads to the expected increase of interfacial volume fraction. On the other hand, Fig. S4 shows that the interfacial volume fraction falls in the order EVF distribution > Fuller distribution > power-law with *a* =3 distribution > power-law with *a* =2 distribution for a constant *V_p_*. Interestingly, according to equation (1), we find that EVF and Fuller distribution functions essentially belong to the power-law distribution corresponding to *a* = 4 and 3.5. That is to say, the interfacial volume fraction increases with the increase of exponent of power-law distribution. In fact, under the same hard particle volume fraction, the larger exponent of power-law distribution generates the more fine particles (see visualization examples depicted in Fig. S5), which results in the larger mean surface area of solid phase in polydisperse particle systems with a larger exponent. As such, the amount of interfaces for wrapping the surface of hard particles with the larger mean surface area is more than that for wrapping the surface of hard particles with the smaller mean surface area. Therefore, from the viewpoint of interfacial volume fraction, we can select a suitable exponent of the power-law gradation to optimize the interfacial volume fraction so as to design an excellent material for the service of industrial engineering.

**S12: Prediction of the effective diffusivity of composite materials with HCSS structures**

We present an application on how such the interfacial property gives rise to the variation of effective diffusivity of composite materials. Our approach incorporates the pioneering features of composite sphere or ellipsoid assemblage models with interfaces developed in previous prominent researches^11-15^. We extend their works by proposing a composite anisotropic rotational symmetric particle assemblage model by the generalized self-consistent (GSC) scheme that is used to predict the effective diffusivity of composite materials. It is essentially stressed that the proposed model can be used to evaluate not only the effective diffusivity, but other effective properties. The detailed description of the proposed model is elaborated as follows:

In accordance with the GSC scheme^14^, we put forward to a composite anisotropic particle model representing heterogeneous composite materials with anisotropic HCSS structured particles. The composite anisotropic particle model is actually a three-phase composite structure that consists of the hard core representing solid inclusion phase, the neighboring shell characterizing interface phase, and matrix, as shown in Fig. S6. It is supposed that the diffusivity of each phase in all directions is uniform, at least, as an average. We divide the three-phase structure into two two-phase cells that the particle phase and the interfacial phase are viewed as the first two-phase structure and another one is naturally composed of matrix and a composite particle phase consisting of hard particle and its adjacent interface, as shown in Fig. S6. Also, each two-phase cell as a basic object of study is implemented to obtain the effective diffusivity of composite materials, since effective properties of two-phase composite media have been broadly researched, and there have been a lot of predominant results that can be incorporated.

We start the first two-phase cell, where particles and interfaces are regarded as hard inclusions and matrix, respectively. We follow our preliminary study^15^ that the effective conductivity of an ergodic two-phase medium is expressed by

$\frac{\mu_{e}}{\mu_{1}}=1+\frac{V_{2}\left(\frac{\mu_{2}}{\mu_{1}}-1\right)\left[1+\left(\frac{2}{3}-L_{2x}\right)\left(1-V_{2}\right)\left(\frac{\mu_{2}}{\mu_{1}}-1\right)\right]}{\left[1+L_{2x}\left(1-V_{2}\right)\left(\frac{\mu_{2}}{\mu_{1}}-1\right)\right]\left[1+\left(1-2L_{2x}\right)\left(1-V_{2}\right)\left(\frac{\mu_{2}}{\mu_{1}}-1\right)\right]}$ (6)

with

$\begin{matrix} L_{2x}=L_{2y}=\left(1-L_{2z}\right)/2 \\ =\left\{\begin{matrix} \frac{s^{-1}(\kappa)}{4([s^{-1}(\kappa)]^{2}-1)^{3/2}}\left(2s^{-1}(\kappa)\sqrt{[s^{-1}(\kappa)]^{2}-1}-\ln\frac{s^{-1}(\kappa)+\sqrt{[s^{-1}(\kappa)]^{2}-1}}{s^{-1}(\kappa)-\sqrt{[s^{-1}(\kappa)]^{2}-1}}\right)\text{ }\kappa>1 \\ 1/3\text{ }\kappa=1 \\ \frac{s^{-1}(\kappa)}{2(1-[s^{-1}(\kappa)]^{2})^{3/2}}\left(\arctan\sqrt{\frac{1}{[s^{-1}(\kappa)]^{2}}-1}-s^{-1}(\kappa)\sqrt{1-[s^{-1}(\kappa)]^{2}}\right)\text{ }\kappa<1 \end{matrix}\right. \end{matrix}$ (7)

where *μ_e_* and *μ*_1_ are the effective conductivities of the medium and matrix, respectively. *V*_2_ and *μ*_2_ are the volume fraction and conductivity of inclusions, respectively. *L*_2_*_x_*, *L*_2_*_y_*, and *L*_2_*_z_* are three components of the depolarization factor of rotational symmetric anisotropic inclusions. Similarly, the above formulas can be mapped into the estimation of effective diffusivity of a two-phase medium by the Nernst-Einstein relationship^16^. To be specific, for the current two-phase cell, *D_c_* / *D_i_* = *μ_e_* /*μ*_1_, and *D_p_* / *D_i_* = *μ*_2_/*μ*_1_, where *D_i_* and *D_p_* are the diffusivities of interfaces and hard anisotropic particles, respectively. *D_c_* is the effective diffusivity of the first two-phase cell, i.e., the diffusivity of the composite particle introduced above. The normalized effective diffusivity of the first two-phase cell can thus be given by

$\frac{D_{c}}{D_{i}}=1+\frac{V_{21}\left(\frac{D_{p}}{D_{i}}-1\right)\left[1+\left(\frac{2}{3}-L_{2x}\right)\left(1-V_{21}\right)\left(\frac{D_{p}}{D_{i}}-1\right)\right]}{\left[1+L_{2x}\left(1-V_{21}\right)\left(\frac{D_{p}}{D_{i}}-1\right)\right]\left[1+\left(1-2L_{2x}\right)\left(1-V_{21}\right)\left(\frac{D_{p}}{D_{i}}-1\right)\right]}$ (8)

Note that the volume fraction of inclusions *V*_21_ in the first two-phase cell should equal to the volume fraction of particles occupying to the whole cell with solid particles and interfaces, that is

$V_{21}=\frac{V_{p}}{V_{p}+V_{si}}$ (9)

where *V_si_* is eventually the volume fraction of interfaces described in this article.

For another two-phase cell, the composite particle phase corresponds to the hard inclusion phase, as shown in Fig. S6. In doing so, *D_c_* / *D_m_* = *μ*_2_/*μ*_1_ and *D_e_* / *D_m_* = *μ_e_* /*μ*_1_, where *D_m_* is the diffusivity of matrix, and *D_e_* is the effective diffusivity of composite materials. In accordance with the component of the composite particle phase illustrated above, the volume fraction of inclusions *V*_22_ in such a two-phase composite cell should be equivalent to the volume fraction of the composite particle phase that is essentially the region of hard inclusions and interfaces occupying to composite materials, that is

$V_{22}=V_{p}+V_{si}$ (10)

Thus, according to equation (10), the normalized effective diffusivity of composite materials with such a three-phase structure is expressed by

$\frac{D_{e}}{D_{m}}=1+\frac{V_{22}\left(\frac{D_{c}}{D_{m}}-1\right)\left[1+\left(\frac{2}{3}-L_{2x}\right)\left(1-V_{22}\right)\left(\frac{D_{c}}{D_{m}}-1\right)\right]}{\left[1+L_{2x}\left(1-V_{22}\right)\left(\frac{D_{c}}{D_{m}}-1\right)\right]\left[1+\left(1-2L_{2x}\right)\left(1-V_{22}\right)\left(\frac{D_{c}}{D_{m}}-1\right)\right]}$ (11)

It is clearly manifested from equations (8)-(11) that the effective diffusivity of composite materials is dramatically dependent on the interfacial property, i.e., the interfacial volume fraction. Interestingly, when we reduce the sphericity of anisotropic particles to the unit, namely, *s* = 1.0, *L*_2_*_x_* = *L*_2_*_y_* = *L*_2_*_z_* = 1/3, the effective diffusivity of composite materials is displayed by

$\frac{D_{e}}{D_{m}}=1+\frac{V_{22}\left(\frac{D_{c}}{D_{m}}-1\right)}{1+\frac{1}{3}\left(1-V_{22}\right)\left(\frac{D_{c}}{D_{m}}-1\right)}$ (12)

with

$\frac{D_{c}}{D_{m}}=\frac{D_{i}}{D_{m}}\left[1+\frac{V_{21}\left(\frac{D_{p}}{D_{i}}-1\right)}{1+\frac{1}{3}\left(1-V_{21}\right)\left(\frac{D_{p}}{D_{i}}-1\right)}\right]$ (13)

Equations (12) and (13) are in line with the results for the effective conductivity reported in previous researches^13,14^. In addition, the present scheme is eventually equivalent to the composite sphere assemblage model^11^. We further consider the thickness of interfaces with zero, namely, *V_si_* = 0 and *D_c_* = *D_p_*, the present result reduces to the well-known Hashin-Shtrikman (HS) lower bound^17^. Therefore, the proposed scheme represents the generalization of the composite sphere assemblage model.

We compare the theoretical prediction model with experimental results of the effective diffusivity from the accelerate chloride ion migration experiment^18^. In the accelerate chloride ion migration experiment, *D_m_* = 2.03 × 10^-12^ m^2^/s, and *D_i_* = 5.745, 3.573 and 3.147 × 10^-12^ m^2^/s at *t* =0.02, 0.04 and 0.05 mm, respectively. Here, the diffusivity of hard inclusions is considered to be 0, i.e., *D_p_* = 0, due to the relative impermeability of hard aggregates in materials. Fig. S7 illustrates comparisons of the experimental results with theoretical prediction results. It can be clearly seen that the theoretical prediction agrees well with the experimental data, which reflects the present scheme considering the interfacial property estimates the effective diffusivity of composite materials with a favourable accuracy.

**Supplementary References**

1. Gao, Y., Schutter, G. D. & Ye, G. Micro- and meso-scale pore structure in mortar in realtion to aggregate content. *Cem. Concr. Res.* **52**, 149–160 (2013).
2. Xu, W. X., Chen, W. & Chen, H. S. Modeling of soft interfacial volume fraction in composite materials with complex convex particles. *J. Chem. Phys.* **140**, 034704 (2014).
3. Xu, W. X. & Chen, H. S. Quantitative characterization of the microstructure of fresh cement paste via random packing of polydispersed Platonic cement particles. *Model. Simul. Mater. Sci. Eng.* **20**, 075003 (2012).
4. Mourzenko, V. V., Thovert, J. F. & Adler, P. M. Percolation of three-dimensional fracture networks with power-law size distribution. *Phys. Rev. E* **72**, 036103 (2005).
5. Xu, W. X., Chen, H. S. & Lv, Z. An overlapping detection algorithm for random sequential packing of elliptical particles. *Physica A* **390**, 2452–2467 (2011).
6. Xu, W. X. & Chen, H. S. Mesostructural characterization of particulate composities via a contact detection algorithm of ellipsoidal particles. *Powder Technol.* **221**, 296–305 (2012).
7. Xu, W. X., Chen, H. S. & Liu, L. Evaluation of mesostructure of particulate composites by quantitative stereology and random sequential packing model of mono-/polydisperse convex polyhedral particles. *Ind. Eng. Chem. Res.* **52**, 6678–6693 (2013).
8. Williams, S. R. & Philipse, A. P. Random packings of spheres and spherocylinders simulated by mechanical contraction. *Phys. Rev. E* **67**, 051301 (2003).
9. Torquato, S. & Jiao, Y. Dense packings of the Platonic and Archimedean solids. *Nature* **460**, 876–879 (2009).
10. Donev, A. *et al*. Improving the density of jammed disordered packings using ellipsoids. *Science* **303**, 990–993 (2004**)**.
11. Nguyen, T. K. & Pham, D. C. Equivalent-inclusion approach and effective medium estimates for elastic moduli of two-dimensional suspensions of compound inclusions. *Phil. Mag.* **94**, 4138-4156 (2014).
12. Benveniste, Y. Exact results for the local fields and the effective moduli of fibrous composites with thickly coated fibers. *J. Mech. Phys. Solids* **71**, 219-238 (2014).
13. Norris, A. N., Sheng, P. & Callegari, A. J. Effective-medium theories for two-phase dielectric media. *J. Appl. Phys.* **57**, 1990-1996 (1985).
14. Benveniste, Y. & Milton, G. W. An effective medium theory for multi-phase matrix-based dielectric composites with randomly oriented ellipsoidal inclusions. *J. Eng. Sci.* **49**, 2-16 (2011).
15. Xu, W. X., Chen, H. S., Chen, W. & Jiang, L. H. Prediction of transport behaviors of particulate composites considering microstructures of soft interfacial layers around ellipsoidal aggregate particles. *Soft Matter* **10**, 627–638 (2014).
16. Moore, W. J. *Physical Chemistry* (4th ed., Prentice-Hall Englewood Cliffs, 1972).
17. Hashin, Z. & Shtrikman, S. A variational approach to the theory of the effective magnetic permeability of multiphase materials. *J. Appl. Phys.* **33**, 3125–3131 (1962).
18. Yang, C. C. & Su, J. K. Approximate migration coefficient of interfacial transition zone and the effect of aggregate content on the migration coefficient of mortar. *Cem. Concr. Res.* **32**, 1559-1565 (2002).
19. Wang, L. B., Wang, X. R., Mohammad, L. & Abadie, C. Unified method to quantify aggregate shape angularity and texture using Fourier analysis. *ASCE J. Mater. Civ. Eng.* **17**, 498-504 (2005).

1. ^1^Institute of Soft Matter Mechanics, College of Mechanics and Materials, Hohai University, Nanjing, China; ^2^State Key Laboratory of Structural Analysis for Industrial Equipment, Dalian University of Technology, Dalian, China; ^3^State Key Laboratory of Simulation and Regulation of Water Cycle in River Basin, China Institute of Water Resources and Hydropower Research, Beijing, China and ^4^School of Materials Science and Engineering, Southeast University, Nanjing, China

   Correspondence: Dr WX Xu or Professor W Chen, Institute of Soft Matter Mechanics, College of Mechanics and Materials, Hohai University, 8 Focheng Road, Nanjing 211100, China. Tel.: +86-25-83786873; Fax: +86-25-83736860.

   E-mail: [xuwenxiang@hhu.edu.cn](mailto:xuwenxiang@hhu.edu.cn) or [chenwen@hhu.edu.cn](mailto:chenwen@hhu.edu.cn) [↑](#footnote-ref-2)
